# Supplementary material for: Using Zebrafish to Elucidate Glial-Vascular Interactions During CNS Development
Source: Front Cell Dev Biol. 2021 Jun 29;9:654338. doi: 10.3389/fcell.2021.654338 (PMC8276133; doi:10.3389/fcell.2021.654338)
Supplement: Supplementary file 1 [file Data_Sheet_1.docx]

Supplementary Material

**
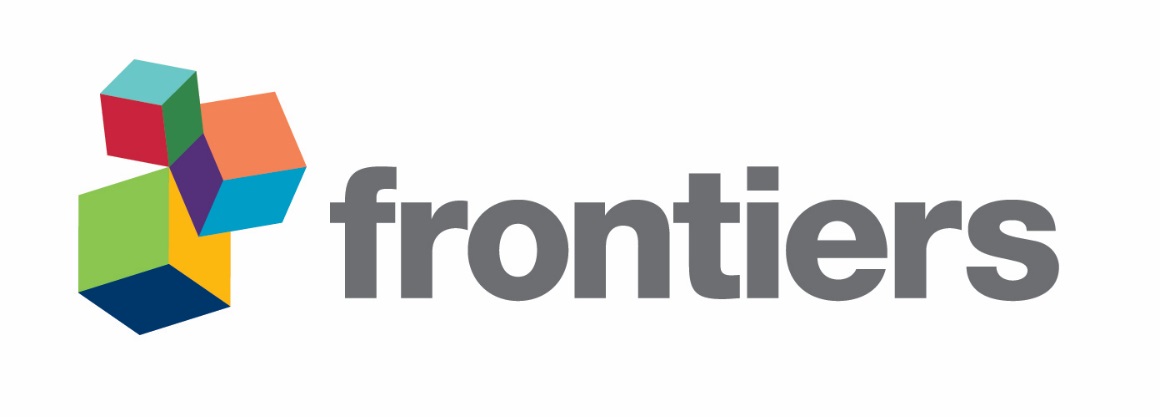
**

**Figure legends:**

**Supplemental Figure 1: Descriptive panel and quantification of glial genes correlates with an increase in *glut1b/slc2a1a* over development.** RT-qPCR from pooled samples revealed that the zebrafish CNS vascular-specific expression of *glut1b/slc2a1a* (**A**) increases over time from 2 to 7dpf after the maternal to zygotic transition. This increase in *glut-1b/slc2a1a* expression parallels a variety of transcripts involved in critical glial homeostasis such as amino acid transport (*glt-1*/*slc1a2b* (**B**) and *glast*/*slc1a3b* (**C**)), channel biology (*aqp4* (**D**) and *kcnj10a* (**E**)), enzymatic activity (*aldh1l1* (**F**) and *glula* (**G**)), and structural composition (*gfap* (**H**)). Each data point contains 3-4 biological replicates, representing 60-80 animals. One-way ANOVAs with multiple comparisons were performed between all time points for each transcript. Error bars represent the standard deviation of the data. *=p<0.05, **=p<0.01,***=p<0.001,****=p<0.0001

**Supplemental Figure 2: XAV939 treatment inhibits Wnt pathway target genes. RT-qPCR was performed on pooled samples (n=4) of either DMSO or XAV939 treated-animals from 3-6dpf. (A)** *lef1* and **(B)** *glut1b* transcripts both decreased after XAV939 treatment. Each data point contains 4 biological replicates, representing 60-80 animals. Error bars represent the standard error of the mean. An unpaired, two-tailed t-test was performed for both RT-qPCR reactions (p<0.05).

**Supplemental Figure 3: Gfap immunohistochemistry in whole mount and thin zebrafish brain sections.** (**A**) Confocal imaging of the brain from a 3dpf vascular transgenic *Tg(flk1:mCherry)* larvae (red) stained for the cardinal glia marker, Gfap (green). Scale bar= 100µm. (**B**) A zoomed in view from the dotted panel in (**A**) showing Gfap cell cytoarchitecture over the vessel. Scale bar= 20µm.

**Supplemental Figure 4: Caspase3 activation after 8-72hpf Metronidazole treatment in *Tg(gfap:nfsB-mCherry)* zebrafish.** Cell death as indicated by activated Caspase3 protein expression was measured through whole-mount immunohistochemistry of (**A**) DMSO vehicle *treated gfap:nfsB-mCherry+* (**B**) MTZ treated *gfap:nfsB-mCherry-* control and (**C**) MTZ treated *gfap:nfsB-mCherry+* siblings. Scale bar=100µm. (**D**) A one-way ANOVA with multiple comparisons was performed between all conditions. Error bars represent the standard deviation of the data. *=p<0.05

**Supplemental Figure 5: *gfap* cell ablation from 1-3dpf causes brain microbleeds and height connection of deep brain vessels.** (**A-C**) Representative brightfield images of (**A**) DMSO vehicle treated *gfap:nfsB-mCherry*+ siblings (**B**) MTZ-treated *gfap:nfsB-mCherry-* control siblings  **(C)** MTZ-treated *gfap:nfsB-mCherry+* siblings. (**C**) 25% of the animals in the MTZ-treated transgenic group developed brain hemorrhages (arrow) n=25 animals. (**D-F**) Representative confocal images of the vasculature (green) from corresponding groups in (A-C). Scale bar=50µm. (**G**) Quantification of posterior communicating segment vessel length in groups mentioned in (**A-C**). n=9 animals. (**H**) Quantification of n=9 animals. A one-way ANOVA with multiple comparisons was performed between all conditions. Error bars represent the standard error of the mean. ***p=<0.001.

**Supplemental Figure 6: CNS angiogenesis is not required for expansion of *gfap* zebrafish glia.** Double transgenic *Tg(kdrl:mCherry;gfap:GFP)* animals were treated for 24h with either DMSO vehicle (**A**) or VEGF inhibitor, AV951 (**B**) and imaged at 2 days post-fertilization. Scale bar=100µm.

**Supplemental Figure 7: Western blot image for Vegfa protein analysis after gfap+ cell ablation.** Samples were loaded in the order of vehicle treated transgenic animals (*gfap:nfsB*)+ (group 1), non-transgenic carrier siblings treated with MTZ (group 2), and transgenic siblings treated with MTZ (group 3), with 6 biological replicates across the groups. A 37kDa band represents the GAPDH loading control and a 20kDa band represents the monomeric version of zebrafish Vegfa. Note the mouse anti- Vegfa antibody used on this blot only recognizes one Vegfa isoform. According to the manufacturer’s product sheet, this corresponds with the Ensembl database entry:ENSDARG00000103542, which is the *vegfaa* paralog on Chromosome 16.

**Supplemental Video 1: *Olig2* glia migrate out as single cells during zebrafish brain development.** Live confocal movie of the *Tg*(*olig2:dsRed;fli1a:eGFP^y1^)* zebrafish line from ~77-99 hours post-fertilization illustrates how individual *olig2* cells (red) extend processes to nearby vessels (green) during CNS development.

**Supplementary Table 1:** **Zebrafish possess orthologs to many common human glia.** Comparison of highly expressed genes from a human transcriptome database (Cahoy et al., 2008) and the corresponding orthologs and annotations in the zebrafish genome.

**Supplemental Table 2: RT-PCR Primer Information.** Primer name, Ensembl genome annotation, sequences, and PCR annealing cycle temperatures from RT-PCR performed in Figures 2 and 6. Bp= base pairs.

**Supplemental Table 1:**

| **Common Name** | **Human Glial Gene** | **Function** | **Zebrafish Glial Gene** | **Annotated in Zebrafish Genome?** | **Ensembl Annotation** |
| --- | --- | --- | --- | --- | --- |
| Glial fibrillary acidic protein | *GFAP* | Intermediate filament protein | *gfap* | Yes | ENSDARG00000025301 |
| Aquaporin-4 | *AQP4* | Water-selective channel that maintains brain water homeostasis | *aqp4* | Yes | ENSDARG00000010565 |
| Phospholipase A2 Group VII | *PLA2G7* | Enzyme that catalyzes the degradation of platelet-activating factors | *pla2g7* | Yes | ENSDARG00000003584 |
| Solute Carrier Family 39 Member 12 | *SLC39A12* | Belongs to subfamily of proteins that structurally resemble zinc transporters | N/A | No | N/A |
| Modulator Of VRAC Current 1 | *MLC1* | Function is unknown; might be an integral membrane transporter | *mlc1* | Yes | ENSDARG00000063026 |
| Type II iodothyronine deiodinase | *DIO2* | Catalyzes production of thyroid hormone (T3) from thyroxine | *dio2* | Yes | ENSDARG00000042112 |
| Urea transporter 1 | *SLC14A1* | Mediates urea transport in erythrocytes | N/A | No | N/A |
| 10-formyltetrahydrofolate dehydrogenase | *ALDH1L1* | Catalyzes conversion of NADP+ and water to tetrahydrofolate, NADPH, and carbon dioxide | *aldh1l1* | Yes | ENSDARG00000077004 |
| Unknown | *CYP4F14* | Metabolizes vitamin E | N/A | No | N/A |
| Aldolase, Fructose-Bisphosphate C | *ALDOC* | Encodes a member of the class I fructose-biphosphate aldolase gene family | *aldocb* | Yes | ENSDARG00000019702 |
| Alpha Tocopherol Transfer Protein | *TTPA* | Regulating, transporting, and facilitating secretion of vitamin E | *ttpa* | Yes | ENSDARG00000027584 |
| Acyl-CoA Synthetase Bubblegum Family Member 1 | *ACSBG1* | Thought to play a central role in brain very long-chain fatty acids metabolism and myelinogenesis | *acsbg1* | Yes | ENSDARG00000062077 |
| Chordin-like protein 1 | *CHRDL1* | May play a role in topographic retinotectal projection and in the regulation of retinal angiogenesis in response to hypoxia | N/A | No | N/A |
| Unknown | *GM266* | Negative regulation of cell migration and Rap protein signal transduction | N/A | No | N/A |
| Solute Carrier Family 4 Member 4 | *SLC4A4* | Sodium bicarbonate cotransporter; maintains intracellular pH | *slca4* | Yes | a) ENSDARG00000013730 b) ENSDARG00000044808 |
| Solute Carrier Family 1 Member 2 | *SLC1A2* | Clears excessive glutamate from synaptic space | *slc1a2* | Yes | a) ENSDARG00000052138 b) ENSDARG00000102453 |
| Solute Carrier Family 25 Member 18 | *SLC25A18* | Transports sugars, organic acids, metal ions, amine compounds, and bile salts | *slc25a18* | Yes | ENSDARG00000070717 |
| Solute Carrier Family 1 Member 3 | *SLC1A3* | High affinity glutamate transporter | *slc1a3* | Yes | a) ENSDARG00000104431 b) ENSDARG00000043148 |
| Coagulation Factor III, Tissue Factor | *F3* | Encodes a cell-surface glycoprotein which forms part of a complex which catalyzes the blood coagulation process | *f3* | Yes | ENSDARG00000041976 |
| Protein Phosphatase 1 Regulatory Subunit 3G | *PPP1R3G* | Glycogen targeting subunit for protein phosphatase 1; involved in regulation of hepatic glycogenesis | N/A | No | N/A |
| Unknown | *CYP4F15* | Monooxygenase involved in metabolic processes | N/A | No | N/A |
| Unknown | *1500005I02RIK* | Functions in protein binding | N/A | No | N/A |
| Frizzled Class Receptor 2 | *FZD2* | Important in Wnt signaling; functions in development | *fzd2* | Yes | ENSDARG00000054438 |
| Unknown | *2900019G14RIK* | Unknown | N/A | No | N/A |
| MER Proto-Oncogene, Tyrosine Kinase | *MERTK* | Receptor tyrosine kinase that is involved in cell migration and differentiation | *mertk* | Yes | ENSDARG00000074695 |
| Villin-2 | *VIL2* | Serves as an intermediate between the plasma membrane and the actin cytoskeleton | N/A | No | N/A |
| Transmembrane Protein 166 | *TMEM166* | Regulates programmed cell death | *tmem166* | Yes | ENSDARG00000067927 |
| Gap Junction Protein Beta 6 | *GJB6* | Encodes one of the connexin proteins found in gap junctions | N/A | No | N/A |
| Hyaluronan And Proteoglycan Link Protein 1 | *HAPLN1* | Used in Integrin pathway and ERK signaling | *hapln1* | Yes | a) ENSDARG00000089769 b) ENSDARG00000068516 |
| Regulatory Factor X4 | *RFX4* | Transcription factor that plays a role in early development | *rfx4* | Yes | ENSDARG00000026395 |
| 3'-Phosphoadenosine 5'-Phosphosulfate Synthase 2 | *PAPSS2* | Bifunctional enzyme which regulates two steps in the sulfate activation pathway | *papss2* | Yes | a) ENSDARG00000071021 b) ENSDARG00000056600 |
| Solute Carrier Family 15 Member 2 | *SLC15A2* | Important in transfer of sugars, bile salts, organic acids, amine compounds, and metal ions. | *slc15a2* | Yes | ENSDARG00000032010 |
| Protein Phosphatase 1 Regulatory Subunit 3C | *PPP1R3C* | Related to basal and insulin-induced glycogen synthesis | N/A | No | N/A |
| Toll Like Receptor 3 | *TLR3* | Plays a role in pathogen recognition and activation of innate immunity | *tlr3* | Yes | ENSDARG00000016065 |
| Acyl-CoA Thioesterase 11 | *ACOT11* | Encodes a member of the acyl-CoA thioesterase family which catalyze the conversion of activated fatty acids to the corresponding non-esterified fatty acid and coenzyme A | *acot11* | Yes | a) ENSDARG00000058229 b) ENSDARG00000042122 |
| ATPase Na+/K+ Transporting Subunit Alpha 2 | *ATP1A2* | Encodes the alpha-2 isoform of the Na(+),K(+)-ATPase, which maintains electrochemical gradients across the plasma membrane | *atp1a2* | Yes | ENSDARG00000010472 |
| Bone Morphogenetic Protein Receptor Type 1B | *BMPR1B* | Encodes a kinase that is involved in endochondral bone formation and embryogenesis | *bmpr1ba* | Yes | ENSDARG00000104100 |
| Unknown | *C030045D06RIK* | Unknown | *c030045d06rik* | No | N/A |
| Proline Dehydrogenase 1 | *PRODH* | Encodes a mitochondrial protein that catalyzes the first step in proline degradation | N/A | No | N/A |
| GLI Family Zinc Finger 3 | *GLI3* | Encodes DNA-binding transcription factors that are mediators of Sonic hedgehog (Shh) signaling | *gli3* | Yes | ENSDARG00000052131 |
| Transmembrane Protein 47 | *TMEM47* | Encodes a protein that regulates cell junction organization in epithelial cells | *tmem47* | Yes | ENSDARG00000057322 |
| SLC9A3 Regulator 1 | *SLC9A3R1* | Encodes a sodium/hydrogen exchanger regulatory cofactor. | *slc9a3r1* | Yes | ENSDARG00000000068 |
| Cystathionine Gamma-Lyase | *CTH* | Encodes a cytoplasmic enzyme in the trans-sulfuration pathway that converts cystathione derived from methionine into cysteine | *cth* | Yes | ENSDARG00000074301 |
| Neurotensin Receptor 2 | *NTSR2* | Encodes a protein that belongs to a G-protein coupled receptor family that activate a phosphatidylinositol-calcium second messenger system | N/A | No | N/A |
| Solute Carrier Family 7 Member 10 | *SLC7A10* | Mediates high-affinity transport of D-serine and several other neutral amino acids | *slc7a10* | Yes | a) ENSDARG00000008100 b) ENSDARG00000051730 |
| Vascular Cell Adhesion Molecule 1 | *VCAM1* | Encodes a cell surface sialoglycoprotein expressed by cytokine-activated endothelium | *vcam1* | Yes | a) ENSDARG00000078201 b) ENSDARG00000062479 |
| Fibroblast Growth Factor Receptor 3 | *FGFR3* | Encodes a member of the fibroblast growth factor receptor (FGFR) family | *fgfr3* | Yes | ENSDARG00000004782 |
| Coiled-Coil Domain Containing 80 | *CCDC80* | Promotes cell adhesion and matrix assembly | *ccdc80* | Yes | ENSDARG00000000002 |
| NTPDase2 | *ENTDP2* | Encodes the type 2 enzyme of the ecto-nucleoside triphosphate diphosphohydrolase family | *entdp2* | Yes | a1) ENSDARG00000035506 a2) ENSDARG00000033953 b) ENSDARG00000044795 |
| Cytochrome B Reductase 1 | *CYBRD1* | Encodes an iron-regulated protein | *cybrd1* | Yes | ENSDARG00000095577 |
| Potassium Voltage-Gated Channel Subfamily E Regulatory Subunit 5 | *KCNE1l* | Encodes a member of a family of single pass transmembrane domain proteins that function as ancillary subunits to voltage-gated potassium channels | N/A | No | N/A |
| Unknown | *BC029169* | Unknown | N/A | No | N/A |
| Unknown | *AI464131* | Unknown | N/A | No | N/A |
| Unknown | *EG328479* | Unknown | N/A | No | N/A |
| Tenascin C | *TNC* | Encodes an extracellular matrix protein; implicated in guidance of migrating neurons as well as axons during development, synaptic plasticity, and neuronal regeneration | *tnc* | Yes | ENSDARG00000021948 |
| TLC Domain Containing 1 | *TlCD1* | Regulates the composition and fluidity of the plasma membrane | *tlcd1* | Yes | ENSDARG00000002391 |
| Endothelial Differentiation Gene 1 | *EDG1* | Encodes a member of a G-protein coupled receptor family which binds the bioactive signaling molecule sphingosine 1-phosphate (S1P) | N/A | No | N/A |
| Cystathionine Beta-Synthase | *CBS* | Acts as a homotetramer to catalyze the conversion of homocysteine to cystathionine, the first step in the transsulfuration pathway | N/A | No | N/A |
| PBX Homeobox Interacting Protein 1 | *PBXIP1* | Interacts with the PBX1 homeodomain protein, inhibiting its transcriptional activation potential by preventing its binding to DNA | N/A | No | N/A |
| Glutamate Ionotropic Receptor NMDA Type Subunit 2C | *GRIN2C* | Encodes a subunit of the N-methyl-D-aspartate (NMDA) receptor | *grin2c* | Yes | ENSDARG00000078149 |
| Unknown | *A730056I06RIK* | Unknown | N/A | No | N/A |
| Alcohol Dehydrogenase Iron Containing 1 | *ADHFE1* | Encodes hydroxyacid-oxoacid transhydrogenase, which is responsible for the oxidation of 4-hydroxybutyrate | *adhfe1* | Yes | ENSDARG00000053518 |
| Angiotensinogen | *AGT* | Encodes pre-angiotensinogen | *agt* | Yes | ENSDARG00000016412 |
| Glycine dehydrogenase | *GLDC* | Encodes the P Protein in the glycine cleavage system | *gldc* | Yes | ENSDARG00000035120 |
| Unknown | *ALC7A2* | Unknown | N/A | No | N/A |
| Unknown | *BC055107* | Unknown | N/A | No | N/A |
| Gap Junction Protein Alpha 1 | *GJA1* | Encodes a protein that is a component of gap junctions | *gja1* | Yes | ENSDARG00000041799 |
| Pyruvate Dehydrogenase Kinase 4 | *PDK4* | Encodes a mitochondrial protein with a histidine kinase domain | *pdk4* | Yes | ENSDARG00000054848 |
| Epidermal Growth Factor Receptor | *EGFR* | Encodes a receptor protein that plays a role in cell proliferation | *egfr* | No | ENDSARG00000013847 |
| Transcription factor SOX-9 | *SOX9* | Encodes a transcription factor that plays a key role in chondrocyte differentiation and skeletal development | *sox9* | Yes | a) ENSDARG00000003293 b) ENSDARG00000043923 |
| Claudin-10 | *CLDN10* | Encodes a member of the claudin family, which are integral membrane proteins and components of tight junction strands | N/A | No | N/A |
| Phospholipase C Delta 4 | *PLCD4* | Encodes a member of the delta class of phospholipase C enzymes | *plcd4* | Yes | a) ENSDARG00000057975 b) ENSDARG00000040684 |
| Inhibitor Of DNA Binding 4, HLH Protein | *ID4* | Encodes a member of the inhibitor of DNA binding (ID) protein family | *id4* | Yes | ENSDARG00000045131 |
| Flavin Containing Dimethylaniline Monoxygenase 1 | *FMO1* | Involved in the oxidative metabolism of a variety of xenobiotics such as drugs and pesticides | N/A | No | N/A |
| Epithelial Membrane Protein 2 | *EMP2* | Encodes a tetraspan protein of the PMP22/EMP family, which regulates cell membrane composition | *emp2* | Yes | ENSDARG00000044588 |
| LON Peptidase N-Terminal Domain And Ring Finger 3 | *LONRF3* | Involved in protein-protein and protein-DNA interactions. | N/A | No | N/A |
| HtrA Serine Peptidase 1 | *HTRA1* | Encodes a member of the trypsin family of serine proteases | *htra1* | Yes | a) ENSDARG00000032831 b) ENSDARG00000014907 |
| Microsomal Glutathione S-Transferase 1 | *MGST1* | Encodes a member of the MAPEG (Membrane Associated Proteins in Eicosanoid and Glutathione metabolism) family | *mgst1* | Yes | 1) ENSDARG00000032618 2) ENSDARG00000022165 |
| Thyroid Hormone Responsive | *THRSP* | Encodes a protein that might play a role in controlling tumor lipid metabolism | *thrsp* | Yes | ENSDARG00000099399 |

**Supplemental Table 2:**

| **Target** | **Ensembl Annotation** | **Primer Sequence 5’—3’** | **Annealing Temperature (°C)** | **Expected Product Size** |
| --- | --- | --- | --- | --- |
| *glt-1/slc1a2b* | ENSDARG00000102453 | (Forward) GACACCAACAGCACCATGTC  (Reverse) CAGGTTCTCCTCCAGACAGC | 52 | 513 bp |
| *glast/slc1a3b* | ENSDART00000131693.3 | (Forward) CTTCGTCATCACTCGCAAAA  (Reverse) CTGTTCCGTCCATGTTGATG | 51 | 197 bp |
| *aqp4* | ENSDARG00000010565 | (Forward) cgttactgtggcaatggttg  (Reverse) ggagaggacgtcagcatagc | 51 | 505 bp |
| *kir4.1/kcnj10a* | ENSDART00000172874.2 | (Forward) TAGACGACAACAGCCCACTG  (Reverse) GCAACCTTGTCGAAGAAAGC | 53 | 244bp |
| *glula* | ENSDARG00000099776 | (Forward) ccagcacgtatcaagctgaa  (Reverse) ccacaccagcagagaactca | 51 | 756bp |
| *aldh1l1* | ENSDARG00000114342 | (Forward) TGCAGTTGCAGAATGAGGAC  (Reverse) TAGAGCCAGTGAAGCCCAGT | 51 | 843bp |
| *s100β* | ENSDARG00000057598 | (Forward) tagagaactgcctgggaacc  (Reverse) accatggtgacgaaggtcat | 52 | 232bp |
| *gfap* | ENSDARG00000025301 | (Forward) GGTCCATGAGGAGGAGATGA  (Reverse) TCCAGCAGCTTCCTGTAGGT | 52 | 507bp |
| *olig2* | ENSDART00000060006.5 | (Forward) TCTGTGCGCAAGCTCTCTAA  (Reverse) ACAACTGGACGGATGGAAAC | 55 | 156bp |
| *slc2a1a/glut1b* | ENSDART00000025414.7 | (Forward) CAACTTGGCATTGTCATTGG  (Reverse) GTGGGCTTTCAGGACAGAAG | 51 | 154bp |
| *vegfaa* | ENSDART00000167719.2 | (Forward) TCCAGGAGTATCCCGATGAG  (Reverse) GCTTTGACTTCTGCCTTTGG | 53 | 548bp |
| *vegfab* | ENSDART00000136229.2 | (Forward) TGTTGGTGGAAATTCAGCAG  (Reverse) CACCCTGATGACGAAGAGGT | 53 | 665bp |
| *Elfa/eef1a1l1* | ENSDARG00000020850 | (Forward) CTTCTCAGGCTGACTGTGC  (Reverse) CCGCTAGCATTACCCTCC | 52 | 358bp |
