# Supplementary material for: Using Zebrafish to Elucidate Glial-Vascular Interactions During CNS Development
Source: Front Cell Dev Biol. 2021 Jun 29;9:654338. doi: 10.3389/fcell.2021.654338 (PMC8276133; doi:10.3389/fcell.2021.654338)

Supplemental Figure 1

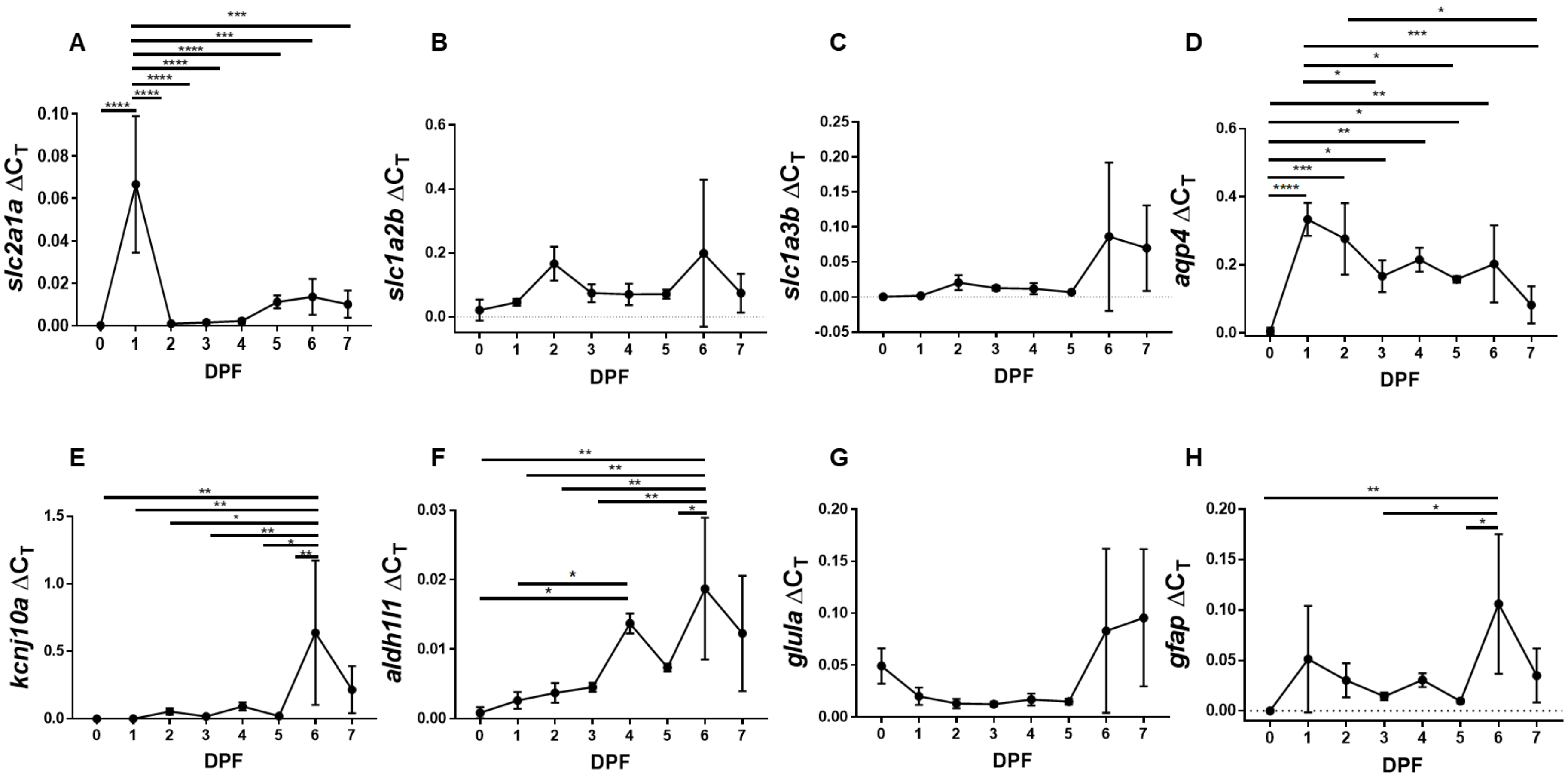

Supplemental Figure 2

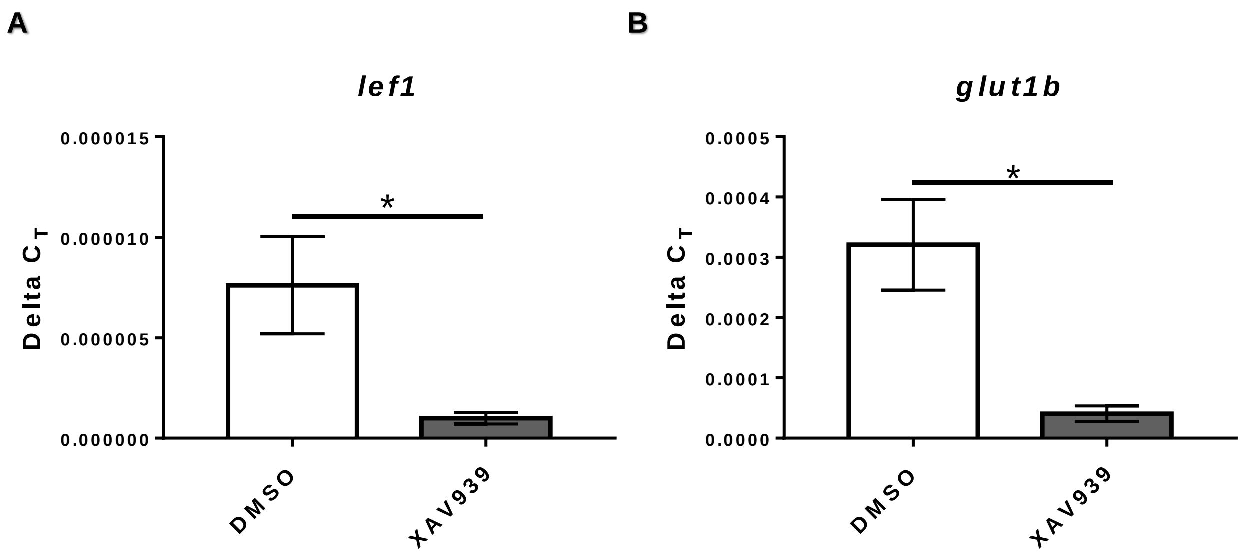

Supplemental Figure 3

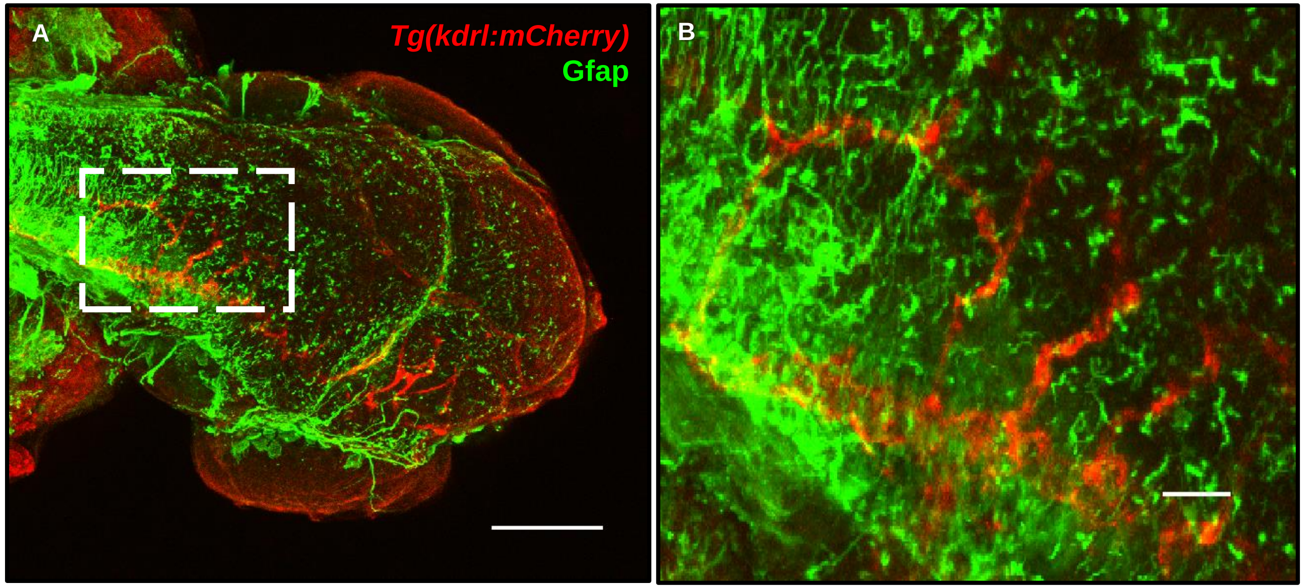

Supplemental Figure 4

C

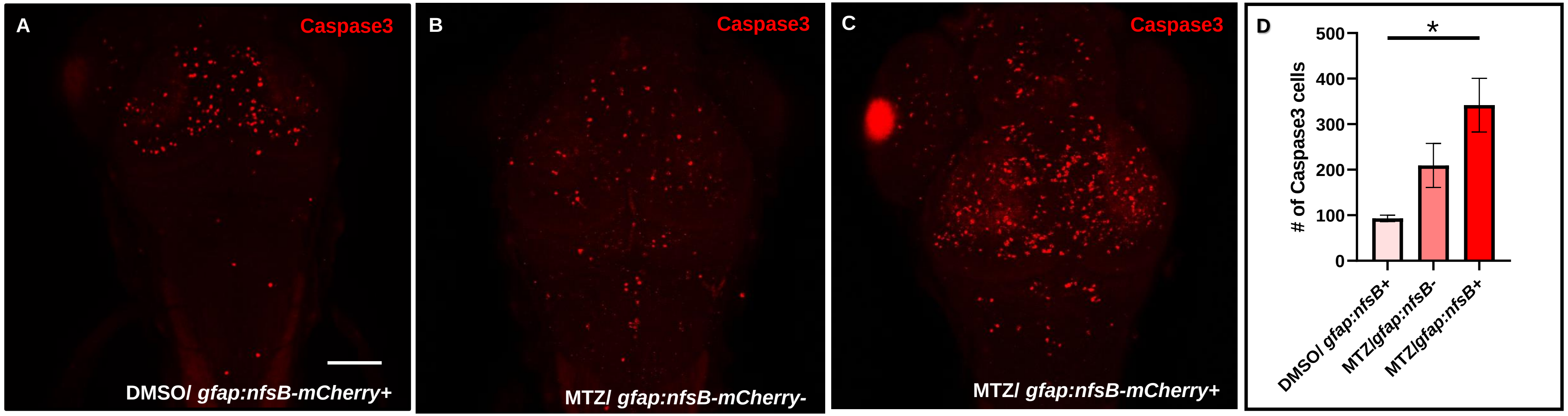

Supplemental Figure 5

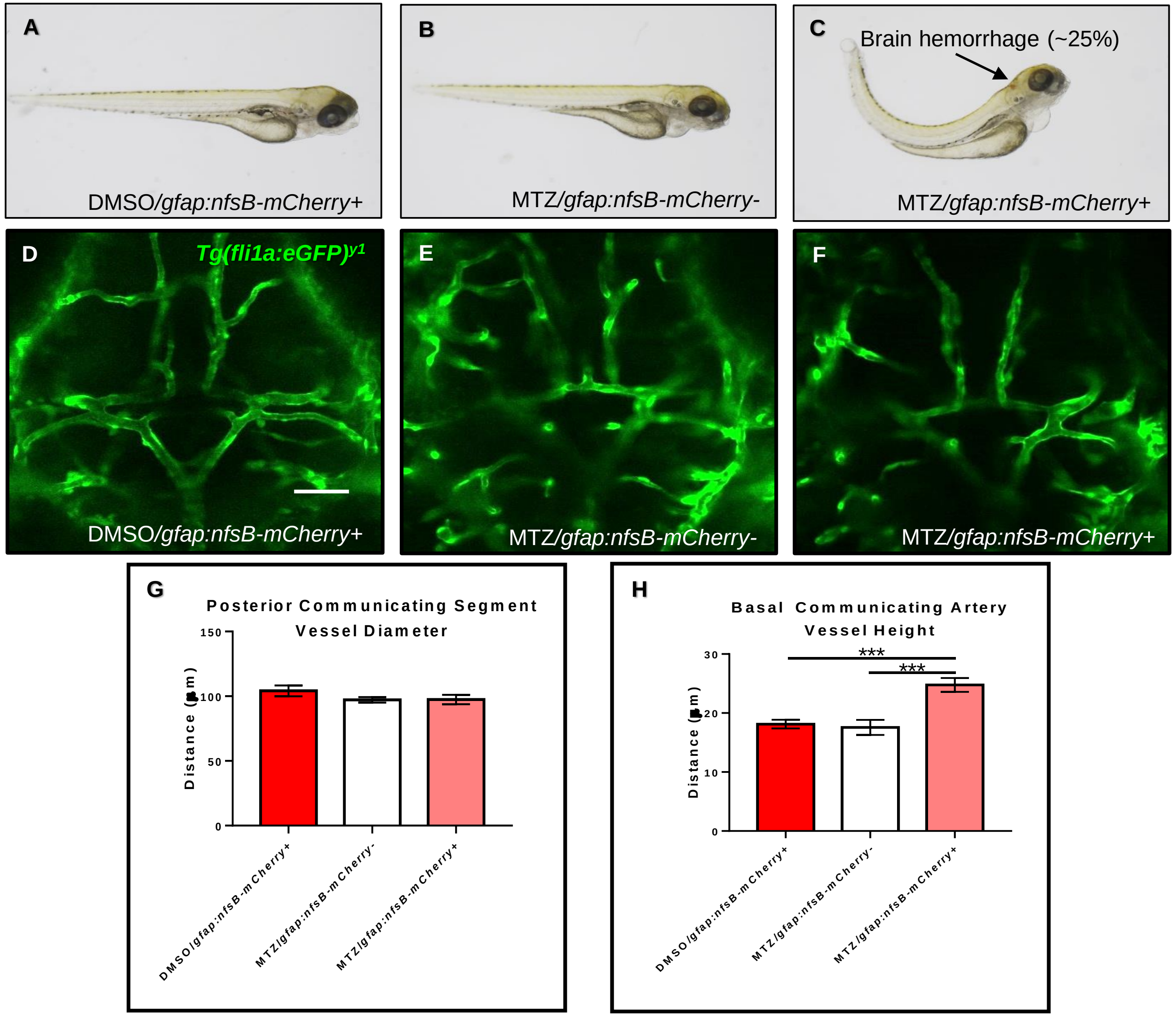

Supplemental Figure 6

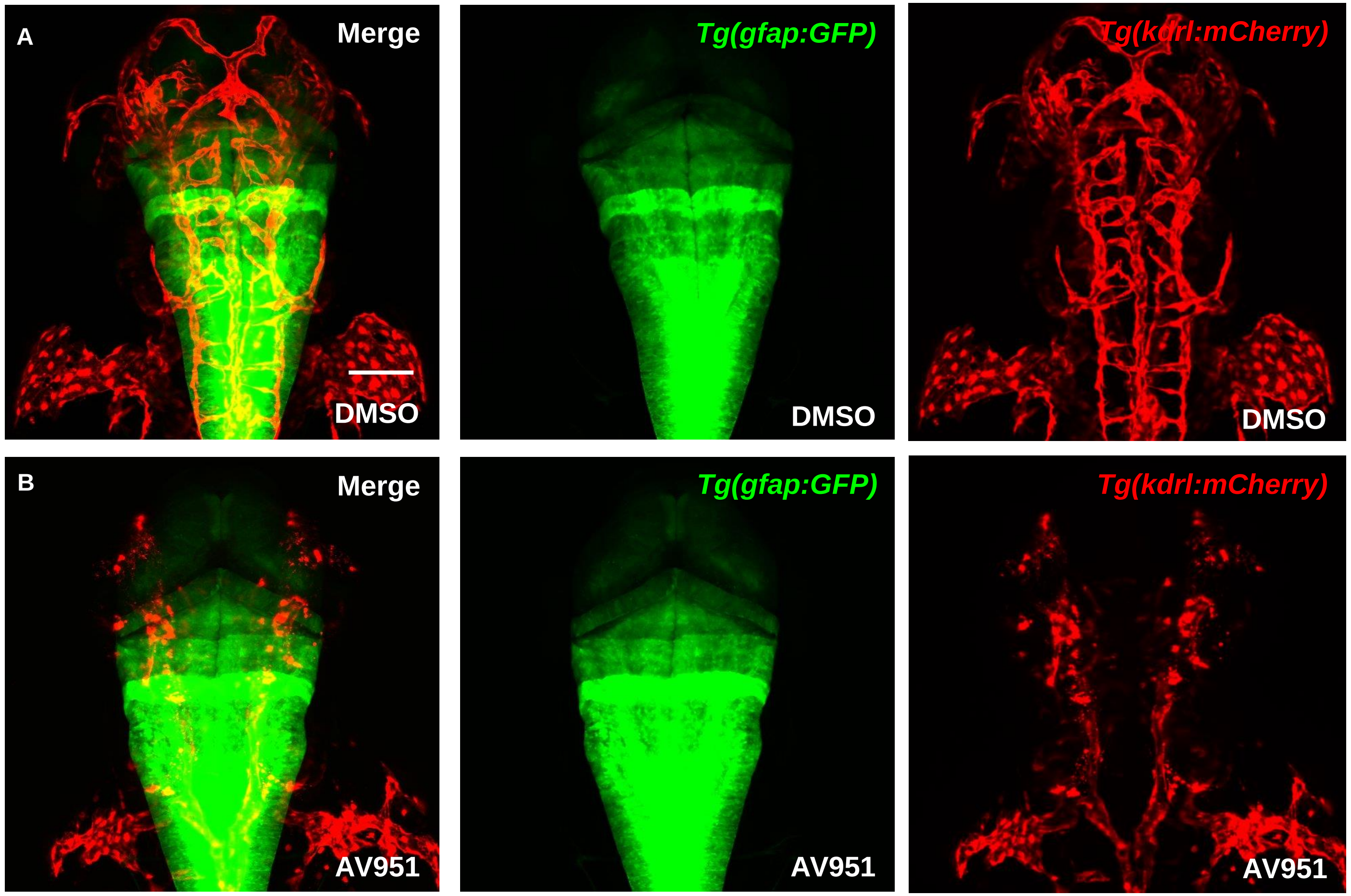

Supplemental Figure 7

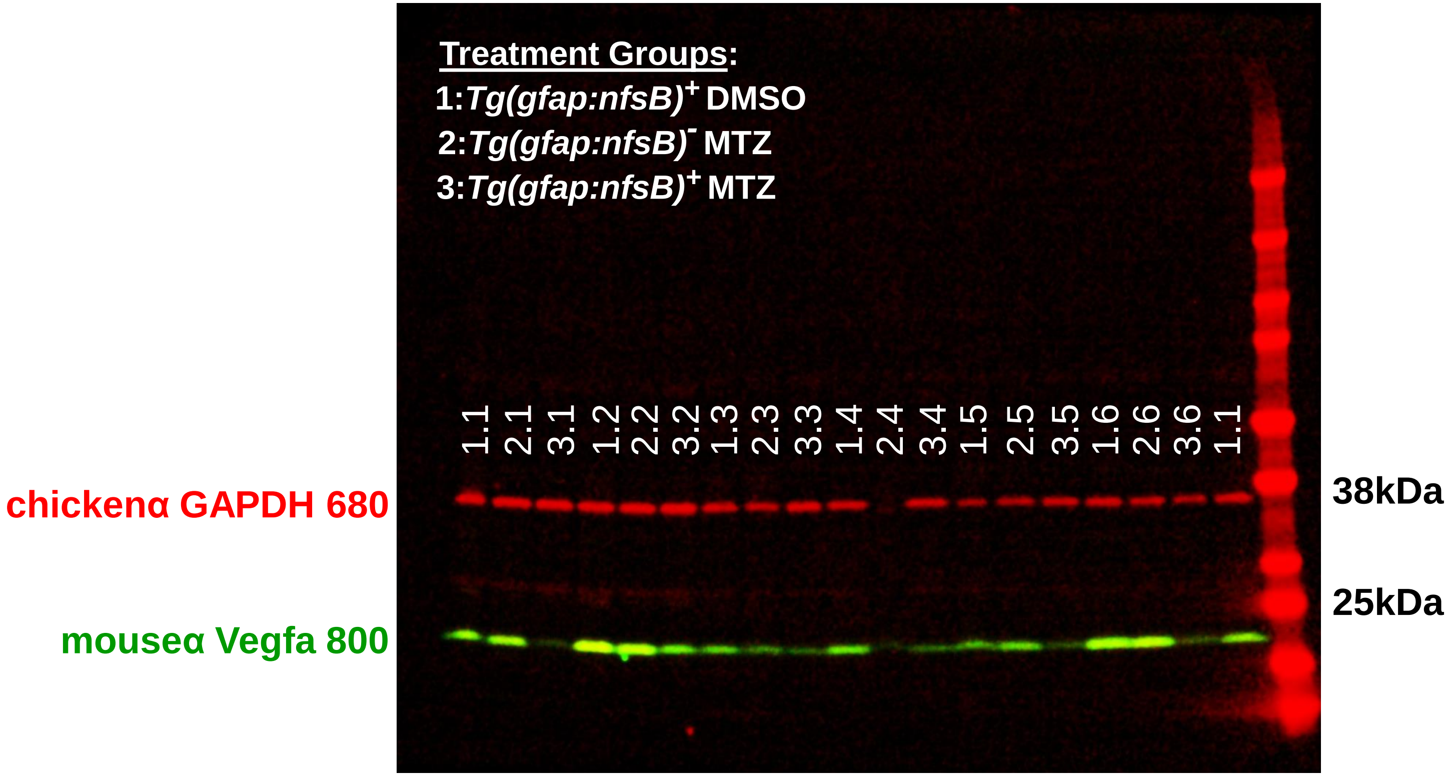

Supplement: Supplementary file 2 [file Image_1.pdf]
